# Genetic Effects on DNA Methylation and Its Potential Relevance for Obesity in Mexican Americans

**DOI:** 10.1371/journal.pone.0073950

**Published:** 2013-09-13

**Authors:** Melanie A. Carless, Hemant Kulkarni, Mark Z. Kos, Jac Charlesworth, Juan M. Peralta, Harald H. H. Göring, Joanne E. Curran, Laura Almasy, Thomas D. Dyer, Anthony G. Comuzzie, Michael C. Mahaney, John Blangero

**Affiliations:** 1 Department of Genetics, Texas Biomedical Research Institute, San Antonio, Texas, United States of America; 2 Menzies Research Institute Tasmania, University of Tasmania, Hobart, Tasmania, Australia; Peninsula College of Medicine and Dentistry, United Kingdom

## Abstract

Several studies have identified effects of genetic variation on DNA methylation patterns and associated heritability, with research primarily focused on Caucasian individuals. In this paper, we examine the evidence for genetic effects on DNA methylation in a Mexican American cohort, a population burdened by a high prevalence of obesity. Using an Illumina-based platform and following stringent quality control procedures, we assessed a total of 395 CpG sites in peripheral blood samples obtained from 183 Mexican American individuals for evidence of heritability, proximal genetic regulation and association with age, sex and obesity measures (i.e. waist circumference and body mass index). We identified 16 CpG sites (∼4%) that were significantly heritable after Bonferroni correction for multiple testing and 27 CpG sites (∼6.9%) that showed evidence of genetic effects. Six CpG sites (∼2%) were associated with age, primarily exhibiting positive relationships, including CpG sites in two genes that have been implicated in previous genome-wide methylation studies of age (*FZD9* and *MYOD1*). In addition, we identified significant associations between three CpG sites (∼1%) and sex, including DNA methylation in *CASP6*, a gene that may respond to estradiol treatment, and in *HSD17B12*, which encodes a sex steroid hormone. Although we did not identify any significant associations between DNA methylation and the obesity measures, several nominally significant results were observed in genes related to adipogenesis, obesity, energy homeostasis and glucose homeostasis (*ARHGAP9*, *CDKN2A*, *FRZB*, *HOXA5*, *JAK3*, *MEST*, *NPY*, *PEG3* and *SMARCB1*). In conclusion, we were able to replicate several findings from previous studies in our Mexican American cohort, supporting an important role for genetic effects on DNA methylation. In addition, we found a significant influence of age and sex on DNA methylation, and report on trend-level, novel associations between DNA methylation and measures of obesity.

## Introduction

DNA methylation is an epigenetic modification that effectively regulates gene expression via gene silencing, and is thus considered an important mechanism in growth and development. Within humans, the DNA methyltransferases, *DNMT1*, *DNMT3a* and *DNMT3b*, regulate DNA methylation, which involves the covalent addition of a methyl group at the C(5) position of cytosine in cytosine-guanine (CpG) dinucleotides [1], [2]. Emerging evidence from both animal and human studies suggest that DNA methylation may be heritable [3]–[7], although the mechanisms of such heritability are speculative.

Both genetic and epigenetic mechanisms can underlie disease development through gene dysregulation, and in many cases act synergistically. DNA methylation changes, at least at the single gene level, have been implicated in most complex diseases, including cancer [2], [8], [9], autoimmune diseases (lupus, multiple sclerosis) [8], [9], psychiatric disorders (bipolar disorder, schizophrenia, autism and depression) [10], obesity [11]–[14], diabetes [15]–[19], atherosclerosis [20], hypertension [21] and cardiovascular disease [22]–[24].

Epigenetic regulation has not been well studied in Mexican Americans, who show a distinct disease profile relative to Caucasian populations. In particular, the prevalence of obesity, diabetes and metabolic syndrome are all elevated among Mexican Americans [25]–[27], which may be mediated, in part, by epigenetic changes. Moreover, obesity is strongly associated with cardiovascular disease, the leading cause of death worldwide and thus merits particular attention. For our study sample, two measures of obesity were taken: body mass index (BMI), which has been historically used to define obesity (i.e. greater than 30 kg/m^2^); and waist circumference, which, as an index for central adiposity, has been shown to be a more accurate indicator of obesity, as well as an independent risk factor for diabetes [28]. To date, several animal and human studies have implicated DNA methylation in obesity [11]–[14], but have primarily focused on global methylation levels or gene-specific DNA methylation rather than a genome-wide approach, or have focused on relatively small sample sizes. In this paper, we report the findings from our pilot study of genome-wide methylation data that investigates: 1) the inherent genetic contribution to epigenetic modifications in a family-based Mexican American cohort; 2) the relationship between DNA methylation and age and sex; and 3) the relationship of DNA methylation with two measures of obesity, BMI and waist circumference.

## Materials and Methods

### Ethics Statement

Written consent was obtained for all individuals in this study. Approval for this study and for the consent process was received from the Institutional Review Board at The University of Texas Health Science Center at San Antonio.

### Population Structure

The San Antonio Family Heart Study (SAFHS) was initiated in 1991 to study the effects of cardiovascular related illness in Mexican Americans [29]. The initial population consisted of 1,431 individuals within 42 extended families. This pilot study assesses DNA methylation profiles of a subset of these individuals (n = 183 successfully profiled subjects from an initial selection of 188 subjects) who represent large founder lineages, chosen to maximize genetic variation. Many of these individuals are related. Pairwise relationships include 196 pairs of 1^st^ degree relatives, 221 pairs of 2^nd^ degree relatives, 401 pairs of 3^rd^ degree relatives, 320 pairs of 4^th^ degree relatives and 39 pairs of 5^th^ degree relatives. The mean age of this population is 42.15 years (standard deviation (sd): 13.74; range 19–75 years) and 55% of individuals are female. Each individual has been assessed for basic anthropometric measures, including those relevant to obesity, waist circumference (n = 182) and BMI (n = 183). In this study, the prevalence of obesity (defined as BMI≥30 kg/m^2^) is 59%, with 86% of the sample falling in the “overweight” category (BMI≥25 kg/m^2^). The average BMI for the cohort is 32.40 kg/m^2^ (sd: 7.40 kg/m^2^), and is 31.21 kg/m^2^ (sd: 6.81 kg/m^2^) for males and 33.38 kg/m^2^ (sd: 7.75 kg/m^2^) for females. The average waist circumference for the cohort is 104.86 cm (sd: 16.14 cm), and is 104.08 cm (sd: 14.91 cm) for males and 105.50 cm (sd: 17.12 cm) for females.

### Methylation Profiling

Using the EZ-96 DNA Methylation™ Kit (Zymo Research Corp, Irvine, CA) and following the manufacturers’ instructions, we performed bisulfite conversion of 1 µg DNA obtained from peripheral blood of an initially selected 188 Mexican American individuals following an overnight fast. We used Illumina GoldenGate technology incorporating the Methylation Cancer Panel I (Illumina, San Diego, CA) to investigate the quantitative level of methylation at 1,505 CpG sites across 807 genes; 28.6% of genes contained only one CpG site, 57.3% contained two CpG sites and 14.1% of genes had three or more CpG sites. Although the CpG sites within this panel were chosen on the basis of their association or potential association with cancer, they fell within genes involved in DNA repair, cell cycle control, differentiation and apoptosis making them suitable for study in disorders other than cancer. The GoldenGate assay was carried out according to manufacturers’ instructions. Throughout the manuscript, CpG sites are annotated as *GENE_Position_Strand*, where *GENE* designates the gene name, *Position* designates the position of the CpG site relative to the transcription start site either within the promoter (*P*) or exon (*E*), and *Strand* designates whether the CpG site is on the forward (*F*) or reverse (*R*) strand.

Initial analysis was carried out using GenomeStudio, applying background normalization by using the signals from built-in controls to minimize the amount of variation in background signals between arrays. DNA methylation for each CpG site is reported as a beta value, which is a score between 0 (completely unmethylated) and 1 (fully methylated). Built-in sample dependent and independent controls were assessed for each sample to look for any outliers. The raw data generated from GenomeStudio is given in Table S1. We excluded three samples with call rates less than 90% (based on detection p-values≥0.05). We excluded two additional samples whose coefficient of variation (based on beta values) was beyond the 97.5^th^ percentile, leaving a total of 183 samples for statistical analyses. Samples used in this study were also genotyped for approximately one million SNPs (see below) as part of a larger study; sex and familial relationships were verified during the genotyping process. Any CpG sites with a call rate less than 0.97 were excluded from analysis (n = 103). The platform used in this study has been shown to discriminate DNA methylation levels (beta values) that differ as little as 17% [30], as such, we excluded probes that showed <0.17 variation in beta values from analysis (n = 355). To account for the non-normal distribution typical of many CpG sites, we used the Lilliefors test for normality [31] at the 0.1 level after FDR correction for multiple testing; non-normally distributed probes were excluded from analysis (n = 641). Any remaining X-linked probes were excluded from analysis (n = 11). Thus, in total, 395 CpG sites were included in subsequent analyses. Polymorphisms within the probe sequence may affect probe binding and therefore bias DNA methylation levels detected. We therefore tested for the presence of 39,706,715 known single nucleotide polymorphisms (SNPs), derived from the 1000 Genomes Project, in each of the 395 probes analyzed.

### High Density SNP Genotyping

Each individual assessed in this pilot study has been genotyped for approximately one million SNP markers using several Illumina genotyping arrays, including the Illumina HumanHap550v3, with HumanExon510Sv1, Human1Mv1 and Human1M-Duov3 (Illumina, San Diego, CA). The Infinium Whole-Genome Genotyping Assay was employed according to manufacturers’ instructions. Genotyping of the full SAFHS cohort included >2,000 samples, which underwent stringent quality control measures, excluding any samples with call rates <90% or that showed discrepancies in either sex or known familial relationships. According to these criteria, all 188 samples that underwent methylation profiling were eligible for genotyping analysis. Data cleaning in the full SAFHS population excluded monomorphic SNPs and those with a call rate less than 95%, those that were monomorphic, those whose minor allele was present in <10 individuals and those with Hardy-Weinberg Equilibrium test statistics of p≤10^−4^ (calculated using SOLAR [32] taking familial relationships into account). A total of 995,320 SNPs were available for analysis of genetic regulation. Allele frequencies were calculated using maximum likelihood estimates in SOLAR [32] and genotypes were checked for Mendelian consistency using Simwalk [33]. Finally, Merlin [34] was used to impute missing genotypes conditional on relatives’ genotypes with a weighted average of possible genotypes being used when an individuals’ genotype could not be inferred with certainty. Raw genotype data for the entire SAFHS cohort will be made available shortly through the Center for Collaborative Genomic Studies on Mental Disorders.

### Statistical Analysis

Our general computer package for statistical genetic analysis, SOLAR [32], was used to estimate heritability, identify genetic variation influencing DNA methylation and determine associations with age, sex and obesity measures. Polygenic regression models of the form: 
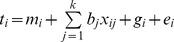
 were used in the analyses where t is phenotypic trait observation on the ith individual, m is the estimated mean trait value, b is the regression coefficient associated with covariate×(total k covariates), g is the polygenic effect and e is the residual error. The polygenic effect g was modeled by including a pairwise kinship matrix with the following kinship coefficients: identical twins, 1; parent-offspring or sibling, 0.5; and grandparent-grandchild, avuncular, half-siblings or double first cousins, 0.25. Further, the kinship coefficients for 3^rd^, 4^th^, 5^th^ and 6^th^ degree relatives are 0.0078, 0.0020, 0.0005 and 0.0001, respectively. The covariates sex, age, age^2^ and their interactions were used in all relevant analyses. Regression terms were estimated for each covariate, the statistical significance of which was assessed using a likelihood ratio test by comparing the log-likelihoods of models in which the covariate effect is estimated to the likelihoods of models in which the covariate effects were constrained to zero. Given that methylation profiling was performed on individuals that were chosen to maximize capture of founder genomes from the largest pedigrees and were optimally chosen for heritability inference, we calculated power to detect additive genetic heritability. We estimated that we have 50% power to detect heritability as small as 0.23 and 80% power to detect heritability as low as 0.36. To detect associations whilst correcting for multiple tests (Bonferroni correction) our power is 80% to detect variants that account for 16.7% of the variation. As such, we present results based on both Bonferroni correction and the less stringent false discovery rate (FDR) of 5%. The tail area-based estimation of FDR was computed using the R program “fdrtool” [35].

We used the BSMAP software package [36] to examine if the autosomal CpG sites that were significantly associated with sex were cross-reactive with the X-chromosome. For this analysis, we used the Hg19 reference genome with the following *in silico* conversions – reference, reverse complement, fully methylated and fully unmethylated. We then aligned the three probe sequences against these four reference genomes.

We employed the “measured genotype” model [37], [38], as implemented in the genome-wide association procedures in SOLAR [39], to investigate the effect of genetic variation on methylation levels at each CpG site. Each of the observed CpG sites was tested as a possible covariate influencing waist circumference or BMI using a regression approach that allowed for non-independence. We also tested for effects of proximal genetic variation, with the assumption that significant findings (correlations) would likely indicate genetic effects. Two CpG sites (*PECAM1_E32_R* and *PECAM1_P135_F*) could not be annotated and were excluded from this analysis. To test for genetic effects on DNA methylation levels, we took the measured-genotype approach to analyses and defined proximal SNPs as those within 100 kb of the flanking region around the genomic location of the CpG site (n = 29,862). According to these criteria, all 393 CpG sites were assessed for genetic effects. For determining the statistical significance of a correlated SNP, the nominal p-value was estimated by comparing the log-likelihoods of a model with and without the SNP. Since we anticipated that many of the SNPs included in these analyses are likely to be in linkage disequilibrium, we used the method suggested by Li and Ji [40] to correct for multiple testing. In this method, the number of independent tests is determined using the eigenvalues of the SNP×SNP correlation matrix and then using a Sidak correction for multiple testing. We conducted these analyses separately for each chromosome and then summated all of the independent tests (correction for 4,209 tests). Additionally, we adjusted for an FDR of 5%.

## Results and Discussion

We have conducted a pilot study to examine the effects of genetic variation on DNA methylation in Mexican Americans, in which these effects, to our knowledge, have not yet been examined. Further, we assessed associations between DNA methylation and age and sex, as well as obesity measures, in order to better understand epigenetic contributions to health.

To ensure DNA methylation levels were not significantly influenced by variation within the probe sequence, we tested for the presence of 39,706,715 SNPs derived from the 1000 Genomes Project. We identified SNPs in 163 of the 395 probes used for analysis (see Table S2); five probes contained three SNPs, 38 probes contained two SNPs and the remaining 120 probes contained a single SNP, three SNPs overlapped with two probes (total of 208 SNPs). Given that most of these SNPs are rare and therefore unlikely to be present in our cohort at a significant frequency, we have highlighted probes containing SNPs with a minor allele frequency (MAF)>5%. We identified SNPs within 24 probes that had a MAF>5%, two of these probes contained two SNPs each. Within these 24 probes, eight contained SNPs within 5 bp of the cytosine molecule of the CpG site and an additional six contained SNPs within 10 bp of the cytosine molecule. Although we included all probes in our analyses, regardless of the presence of SNPs, in the following text (and shown in Table S2), we have highlighted any results that are likely to be influenced by the inclusion of such probes. Only probes that showed significant heritability or genetic effects contained SNPs with a MAF>5%.

### DNA Methylation is Heritable

Of the 395 high quality CpG sites examined in this study, 95 (∼24%) exhibited nominally significant (p<0.05) heritability, 28 (∼7%) were heritable at an FDR of 5% and 16 (∼4%) exhibited statistically significant heritability after Bonferroni correction for multiple testing (p<1.27×10^−4^; Table S3). Table 1 shows all sites that are significantly heritable. Across all loci, the mean heritability was 14%, but it was 36% for the 95 CpG sites that showed nominal evidence for heritability, 52% for the 28 sites that were heritable at an FDR of 5% and was 59% for the 16 CpG sites that were significantly heritable after Bonferroni correction. One of the CpG sites that was found to be heritable (*LTA_E28*) was within a probe sequence that contained a SNP with a MAF of 35%, 19 bp from the CpG site (see Table S2). Although a SNP at this distance is unlikely to affect probe binding, alternative strategies to determine the methylation status of this CpG site may be warranted. An additional five probes that were associated with heritability contained one or two SNPs, although the MAF of these was ≤1% and they are therefore unlikely to influence results due to the presence of perhaps only one or two copies in our cohort.

**Table 1:** Heritability and genetic regulation of DNA methylation.

CpG Site#	Heritability		Genetic effects					
	h^2^	P-value	Number of associations	Most significant SNP	Genotypes (MAF)*	Distance from CpG site (bp)	Beta	P-value
@ABL2_P459_R	0.223	3.87×10^−2^	8	rs1318056	C/G (0.05)	−87,050	1.069	**2.23**×**10^−6^**
*ACVR1_E328_R*	0.494	**9.50**×**10^−6^**	0	rs7595478	C/T (0.13)	−88,318	0.442	1.29×10^−3^
*ALOX12_E85_R*	0.552	**3.20×10^−5^**	9	rs10852889	T/C (0.38)	1,356	−0.911	**5.51×10^−21^**
*ALOX12_P223_R*	0.599	**1.38×10^−5^**	10	rs10852889	T/C (0.38)	1,664	−0.997	**3.56×10^−26^**
*AXL_P223_R*	0.534	**3.00×10^−7^**	7	rs338585	C/T (0.39)	−12,785	0.741	**1.18×10^−16^**
*CDK10_P199_R*	0.000	5.00×10^−1^	1	rs459920	T/C (0.30)	−22,052	0.493	**1.88×10^−6^**
*COL1A2_P407_R*	0.220	2.05×10^−2^	2	rs3763469	A/G (0.13)	−1,991	1.243	**1.22×10^−20^**
*DNAJC15_E26_R*	0.283	2.77×10^−2^	12	rs17553284	C/T (0.17)	318	0.898	**1.38×10^−10^**
*DSC2_E90_F*	0.434	**4.16×10^−5^**	0	rs1313586	A/G (0.25)	−95,323	0.320	3.59×10^−3^
@GDF10_P95_R	0.233	5.97×10^−3^	2	rs11595733	A/G (0.10)	2,563	0.994	**1.85×10^−8^**
*GNMT_P197_F*	0.094	2.11×10^−1^	3	rs2395943	G/A (0.38)	12,370	0.514	**3.74×10^−7^**
*GSTM1_P266_F*	0.732	**3.00×10^−7^**	11	rs3754446	G/T (0.53)	23,065	−1.016	**1.80×10^−30^**
@HHIP_P307_R	0.425	9.85×10^−3^	27	rs7680661	A/G (0.24)	−1,750	0.944	**1.01×10^−14^**
@HHIP_P578_R	0.073	3.32×10^−1^	6	rs2883154	C/T (0.24)	−34,750	0.662	**5.97×10^−10^**
*HOXA5_P1324_F*	0.726	**2.99×10^−5^**	0	rs10951154	T/C (0.14)	−49,297	−0.455	2.46×10^−3^
*HPN_P823_F*	0.549	**1.08×10^−5^**	1	rs2278996	A/C (0.09)	194	−0.960	**3.10×10^−9^**
*HS3ST2_E145_R*	0.591	**4.36×10^−5^**	0	rs210090	T/G (0.30)	24,931	−0.309	7.70×10^−3^
*IL16_P93_R*	0.436	3.40×10^−3^	23	rs7182786	A/G (0.40)	−843	−0.881	**5.91×10^−23^**
*INS_P248_F*	0.175	5.15×10^−2^	6	rs7111341	C/T (0.21)	30,494	−0.719	**6.05×10^−8^**
*IRF5_E101_F*	0.467	**3.83×10^−5^**	1	rs6955705	T/C (0.28)	−20,725	−0.750	**8.45×10^−11^**
@LTA_E28_R	0.566	**2.31×10^−5^**	29	rs2516390	A/G (0.46)	−10,238	0.788	**1.09×10^−16^**
*MEST_P62_R*	0.307	5.87×10^−2^	14	rs7803211	C/T (0.28)	28,617	0.769	**2.61×10^−12^**
*MET_E333_F*	0.626	**3.30×10^−8^**	1	rs184953	C/A (0.19)	−862	0.903	**6.38×10^−12^**
*NPR2_P618_F*	0.674	**2.53×10^−10^**	13	rs2236293	G/A (0.49)	49,995	0.570	**7.67×10^−8^**
*PLAT_P80_F*	0.417	2.72×10^−4^	1	rs2020919	T/C (0.05)	−65	−0.988	**1.17×10^−5^**
*PLSCR3_P751_R*	0.516	3.25×10^−4^	6	rs4796399	A/C (0.46)	−84,900	−0.480	**8.65×10^−7^**
*PSCA_E359_F*	0.053	3.39×10^−1^	1	rs12155758	G/A (0.39)	3,613	−0.547	**3.16×10^−7^**
*PTK7_E317_F*	0.848	**5.00×10^−7^**	0	rs34574340	C/T (0.01)	−30,267	1.133	2.75×10^−2^
*SEMA3B_P110_R*	0.567	**1.65×10^−5^**	0	rs2518796	A/G (0.26)	−97,855	−0.251	4.01×10^−2^
*SLC22A3_P634_F*	0.420	2.77×10^−3^	11	rs487060	C/T (0.38)	5,668	−0.533	**1.65×10^−7^**
*SNCG_P53_F*	0.549	**1.10×10^−6^**	1	rs1800373	G/T (0.46)	74	−0.441	**1.15×10^−5^**
*SPARC_P195_F*	0.390	1.07×10^−2^	6	rs17112187	C/A (0.14)	−6,974	1.194	**9.78×10^−22^**
*SPP1_P647_F*	0.000	5.00×10^−1^	2	rs6813526	T/C (0.27)	−1,920	0.569	**8.46×10^−7^**

P-values that are significant after correction for multiple testing are given in **bold.**

# CpG sites are annotated according to *GENE_Position_Strand*, as outlined in the methods section.

@Indicates probe sequences containing SNPs with a MAF>5%.

* MAF: Minor allele frequency; minor allele is reported second.

Several recent studies have examined the heritability of DNA methylation, finding it to be highly variable for individual CpG sites. Using the Illumina GoldenGate platform Boks, et al, [41] estimated the mean heritability to be 22% (peripheral blood cells) across 430 tested loci and ∼3% of loci were found to be significantly heritable (applying Bonferroni correction). Our results using this same platform revealed a similar, although slightly higher, percentage of significantly heritable sites. Using the Illumina HumanMethylation27 platform that assesses ∼27,000 CpG sites, mean heritability has previously been estimated at: 18% in peripheral blood cells [42]; 12%, 7% and 5% in human umbilical vein epithelial cells, cord blood mononuclear cells and placenta, respectively [43]; and 3% in brain [44]. Our overall mean heritability (14%) is within the range of these study estimates and was determined based on a slightly larger sample size and family data rather than twin or singleton data. Recently, highly variable DNA methylation patterns were identified across three different neonatal tissues and no compelling evidence for a common set of highly heritable DNA methylation variants was seen [43], suggesting that the tissue type analyzed plays a role in heritability estimates.

Given the overlap in the platform and quality control strategies employed, we compared our heritability findings to those of Boks and colleagues, who examined heritability in a twin dataset using 430 CpG probes [41]. Of the 395 probes that were deemed of suitable quality for heritability analysis in our study, only 232 of these (59%) were assessed in the study by Boks, et al, reflecting differential DNA methylation profiles across populations, which may represent ethnic-specific differences or may be due to small cohort sizes and subsequent underpowered analyses. Of the 16 sites we identified as heritable (after Bonferroni correction), five were also identified as heritable in the previous study, one site was nominally significant, seven sites were not assessed and three sites were not heritable in the previous study. In particular, DNA methylation at *NPR2_P618*, *MET_E333_F*, *AXL_P223*, *HPN_P823_F* and *HOXA5_P1324_F* were found to be heritable in both studies. Of the CpG sites that were assessed in both studies, evidence for heritability of DNA methylation at *IRF5_E101_F*, *DSC2_E90_F* and *HS3ST2_E145_R* was found in our study only, which may represent population-specific DNA methylation changes driven by heritable factors. We should also note that both our study, and that by Boks and colleagues, assessed heritability in small cohorts (183 and 82 individuals, respectively) and may therefore be underpowered to accurately identify heritable factors influencing DNA methylation or may be prone to the detection of false positive results. A recent study by Breton, et al, [45] presented contrasting findings for the *AXL_P223* locus, suggesting that DNA methylation was not heritable but was highly influenced by environmental factors, however this too was performed in a small sample set of only 36 twins and as such is likely limited in its power to detect heritability. Nonetheless, given the overlap for several CpG sites across our study and that by Boks, et al, the case for DNA methylation being a heritable trait is becoming stronger and the application of heritability analysis to larger cohorts will ultimately be highly informative.

### Proximal Genetic Variants Influence DNA Methylation

We studied the association of a total of 29,862 SNPs within the 100 Kb flanking region of 393 CpG sites (two sites were excluded due to inability to be annotated), applying a Sidak correction for 4,209 independent tests. In total, 214 statistically significant correlations were detected across 27 CpG sites (p<1.19×10^−5^, 6.9%; Table S4); the most significant association for each CpG site is shown in Table 1. All 16 CpG sites that were significantly heritable also showed at least nominal evidence of being influenced by genetic effects, although several sites that showed nominal evidence for heritability were not found to be regulated by genetic variation (Table S3). We should note that four CpG sites influenced by genetic effects were within probes that also contained SNPs with a MAF>5% (*ABL2_P459*, *GDF10_P95*, *HHIP_P307*, *LTA_E28*; see Table S2), three of these had SNPs within 5 bp of the CpG site. It is therefore possible that the genetic effects seen for these CpG sites are in fact due to linkage disequilibrium between genetic variants influencing probe binding rather than a true biological effect. The SNP contained within the *LTA_E28* probe (rs2239704) was directly tested for association with DNA methylation levels and found to be significantly associated (p = 1.09×10^−16^; see Table S4). An additional eight probes contained between one and three SNPs, although all had a MAF≤3% and are therefore unlikely to influence the results.

Recent genome-wide analyses have indicated that DNA methylation may indeed be tightly regulated by genetic factors. In fact, across studies 4–9% of CpG sites show evidence of genetic effects by SNPs within 50 kb-1 Mb of the site [42], [46], [47]. Applying stringent criteria for the localization of proximal genetic variants (100 kb) and correction for multiple testing (Li and Ji’s correction for 4,209 independent CpG site/SNP association tests; adjusted alpha value 1.18×10^−5^), we found that DNA methylation levels within 6.9% of CpG sites tested were correlated with one or more SNPs. Although this is well in the range of the studies reported above, it is slightly higher than what was reported in the study by Boks, et al, (4%) using the same DNA methylation platform [41]. This may be explained by the smaller sample size (n = 91) they used, a smaller set of SNPs to assess (∼550,000 compared to ∼1,000,000 in our study) and a larger defined region for regulatory variation (1 Mb versus 100 kb in our study), which increases the number of statistical tests that have to be corrected for, thereby reducing power to detect associations. In fact, Quon and colleagues systematically varied the window size to detect regulation using 10 kb, 50 kb, 100 kb, 500 kb and 1 Mb windows surrounding the CpG site to identify the optimum window for detection of heritable sites [44]. Their results showed that a window size of 50 kb yielded the highest number of heritable methylation loci, although similar numbers were seen for the 100 kb window, which we used in our study.

Once again, we compared our regulatory findings to those presented by Boks, et al, who used the same platform but in a singleton dataset, examining 512 probes [41]. Of the 393 high quality probes assessed in our study, only 155 (39%) were also assessed in the study by Boks and colleagues. Of the 23 CpG sites that showed evidence for genetic effects in our study, seven were previously identified as regulated by genetic variation in the study by Boks, et al, (defined as FDR<0.05; *ALOX12_E85*, *ALOX12_P223*, *GNMT_P197_F*, *HPN_P823*, *MET_E333*, *NPR2_P618_F* and *SLC22A3_P634_F*). In all cases where the same SNP was used to test for regulatory effects, the direction of effect was identical in both studies.

In total, all of our significantly heritable CpG sites (p<1.27×10^−4^) showed at least nominal evidence for genetic effects; 10 of the 16 heritable CpG sites (63%) showed strong evidence for regulation by genetic variation. For nominally heritable CpG sites (p<0.05, n = 94 assessed for genetic effects), 21% showed significant evidence for genetic effects and a further 65% showed nominal evidence for genetic effects; only 14% of nominally heritable sites showed no evidence of genetic effects (see Table S3). Although it is tempting to suggest that heritability associated with DNA methylation is derived solely through association with regulatory variants, the fact that not all nominally heritable sites are regulated by such variants suggests that at least a portion of the heritability may be due to other factors. Based on *in silico* SNP analysis and animal studies, Kaminsky and colleagues suggest that the molecular mechanisms driving heritable epigenetic differences may not be limited to sequence differences [6]. Further, evidence from mouse studies suggest that epigenetic marks may not be completely erased, resulting in transgenerational inheritance of epigenetic modifications [3], [4].

### Age and Sex Effects on DNA Methylation Profiles

We also tested for effects of age and sex on DNA methylation profiles and found substantial evidence for both; Table 2 displays CpG sites significantly associated with age and sex after Bonferroni correction. DNA methylation at 88 of the 395 CpG sites (∼22%) showed nominally significant associations with age (p<0.05), 24 CpG sites (∼6%) were significant at an FDR of 5% and six CpG sites (∼2%) were significant after Bonferroni correction for multiple testing (Table S5). Four of the probes analyzed contained one or two SNPs, however these were all at a MAF≤1% and would be unlikely to influence the results (see Table S2). Only one (*CARD15_P302*) of the six associations that we identified was also identified as significant by Boks and colleagues [41], with one site nominally significant (p<0.05), and the other four not assessed. For sex, 50 CpG sites (∼13%) showed nominally significant associations (p<0.05) and three (∼1%) of these were significantly associated with sex after Bonferroni correction for multiple testing and at an FDR of 5% (Table S6). One of the significantly associated probes contained a SNP with a MAF of 1% (see Table S2), which is unlikely to influence the results. Similar to our results with age, the study by Boks, et al, [41] did not assess any of the three CpG sites that we identified as significantly associated with sex.

**Table 2:** Significant age and sex associations with DNA methylation.

CpG Site#	Beta*	P-value
**Age**		
*FZD9_E458_F*	0.039	**8.06**×**10^−8^**
*MYOD1_E156_F*	0.035	**2.75**×**10^−6^**
*HS3ST2_E145_R*	0.033	**3.12**×**10^−6^**
*CARD15_P302_R*	−0.029	**6.70**×**10^−5^**
*CDH11_P354_R*	0.030	**9.29**×**10^−5^**
*IL17RB_E164_R*	0.029	**1.12**×**10^−4^**
**Sex**		
*CASP6_P201_F*	1.369	**2.81**×**10^−18^**
*RET_seq_54_S260_F*	−0.966	**1.51**×**10^−7^**
*HSD17B12_P97_F*	0.756	**7.55**×**10^−5^**

P-values that are significant after correction for multiple testing are given in **bold.**

# CpG sites are annotated according to *GENE_Position_Strand*, as outlined in the methods section.

* Note: a positive value for beta indicates increased methylation is associated with increased age and females.

Recent genome-wide studies have identified a number of age related differentially methylated regions with 78–98% of associated CpG sites showing positive associations [42], [48], [49]. Similarly, of the six CpG sites that we found to be associated with age, five showed a positive association (83%); only the *CARD15_P302* site showed a negative direction of effect (i.e. decreased methylation was associated with increased age), and this was the same direction of effect that was observed in the study by Boks, et al [41]. Positive associations were seen for two CpG sites within genes that have previously been implicated with age in one or more genome-wide methylation studies (*FZD9* and *MYOD1*) [42], [49], [50]. Of these, methylation within *MYOD1* was associated with age in all three previously published studies, as well as in our own. Although two studies were performed in peripheral blood cells, the third assessed DNA methylation that was consistently associated with aging across four brain regions, suggesting that the DNA methylation changes associated with the *MYOD1* gene may be general rather than tissue-specific. In addition to replicating results obtained from these genome-wide studies, we have identified new loci, possibly specific to the Mexican American population, whose DNA methylation status may be related to age. Due to the small number of samples assessed in our study however, such findings would need to be verified.

Despite evidence for sexual dimorphism in a number of diseases, a recent genome-wide association study meta-analysis [51] failed to reveal any genome-wide significant common SNP differences between men and women, which suggests that at least some sex-based differences in disease may be driven more so by either rare variants or epigenetic effects. It is clear that DNA methylation plays a sex-specific role during fetal germline progression [52] and a recent genome-wide methylation study identified DNA methylation differences between the two sexes, although these differences were subtle [53]. Similarly, we identified significant associations between sex and DNA methylation at three CpG sites, each of which has been at least loosely implicated in sex-specific functions of disease. We found highly significant DNA methylation changes at *CASP6_P201*, showing increased methylation in women. Although a sex-specific role has not yet been defined, an early study in rats suggests that caspase-6 is involved in the control of apoptotic processes at estrus following treatment with the sex-hormone 17β-estradiol [54]. Increased methylation at *HSD17B12_P97*, which encodes a sex steroid hormone, was also seen in females. *HSD17B12* has been shown to be involved in the catalytic conversion of oestrone to estradiol and has also been implicated in fatty acid synthesis (reviewed in [55]), which shows sex-specific differences (reviewed in [56]). DNA methylation at *RET_seq_54_S260* was increased in males, and although specific sex effects have not been noted, genetic variants within this gene have been shown to have different effects in males and females, and parent of origin effects involving such variants have been noted in Hirschsprung disease [57], [58]. It is important to note that some level of cross-reactivity with sex chromosomes has been reported in ∼6–10% of the autosomal probes used in some of the higher-throughput Illumina platforms (i.e. 27 k and 450 k methylation chips), due to high sequence homology [59], [60]. This may generate spurious signals that overestimate the effect of DNA methylation on sex and as such, DNA methylation changes that are determined by probe-based methods and are associated with sex should be interpreted with caution. Using BSMAP [36], we observed that none of the three significant sex-associated CpG sites identified in our study (*CASP6_P201, HSD17B12_P97* or *RET_seq_54_S260*) could be mapped to the X-chromosome, using the reference, reverse complement, bisulfite converted fully methylated and bisulfite converted fully unmethylated genomes. Further, given some of the plausible biological roles discussed above, we believe that this study provides additional evidence for a potential role of DNA methylation in sex.

### Methylation Profiles show Nominal Association with Obesity Measures

To investigate a potential role of DNA methylation in disease, we looked for association with the obesity measures, waist circumference and BMI. In particular, waist circumference, which measures central obesity, is considered a stronger indicator of risk for diabetes and cardiovascular disease than BMI [28]. Table 3 shows the ten most significant associations with waist circumference, although none of these are within the threshold for multiple testing correction (p<1.27×10^−4^). In total, we identified 27 and 12 nominally significant (p<0.05) associations between DNA methylation and waist circumference and BMI, respectively (Table S7). Several nominally associated CpG sites are in genes that have a known or plausible role in obesity and diabetes.

**Table 3:** Most highly significant associations between DNA methylation and obesity measures.

CpG Site#	Waist Circumference		Body Mass Index (BMI)	
	Beta*	P-value	Beta*	P-value
*SEPT5_P441_F*	−0.238	5.47×10^−4^	−0.146	3.04×10^−2^
*LMTK2_P1034_F*	−0.193	2.84×10^−3^	−0.117	6.46×10^−2^
*SFTPB_P689_R*	−0.203	4.00×10^−3^	−0.162	1.79×10^−2^
*ARHGAP9_P518_R*	−0.180	5.96×10^−3^	−0.086	1.78×10^−1^
*KRT5_P308_F*	−0.187	6.42×10^−3^	−0.073	2.83×10^−1^
*ZIM2_P22_F*	−0.170	8.75×10^−3^	−0.066	2.97×10^−1^
*PEG3_E496_F*	−0.168	9.45×10^−3^	−0.151	1.62×10^−2^
*CD81_P272_R*	−0.173	1.17×10^−2^	−0.150	2.31×10^−2^
*MEST_P62_R*	−0.174	1.29×10^−2^	−0.137	4.41×10^−2^
*TSP50_E21_R*	−0.168	1.50×10^−2^	−0.059	3.79×10^−1^

Note: all significance values are nominal.

# CpG sites are annotated according to *GENE_Position_Strand*, as outlined in the methods section.

* A positive value for beta indicates increased methylation is associated with increased obesity measures (i.e. increased waist circumference or increased BMI).

Methylation levels of a CpG site within *MEST*, were found to be negatively associated with both waist circumference (nominal p = 1.29×10^−2^) and BMI (nominal p = 4.41×10^−2^). *MEST* is well known to be involved in obesity and glucose metabolism. Gene expression increases have been shown in white adipose tissue of mice with diet-induced and genetically caused obesity/diabetes [61] and in humans, decreased methylation of *MEST* has been shown in morbidly obese individuals as well as in newborns of mothers who had gestational diabetes [62]. We also identified a negative association between DNA methylation within *NPY* and waist circumference (nominal p = 2.96×10^−2^), but not with BMI. *NPY* is a hypothalamic peptide which regulates feeding behavior and energy homeostasis and has been proposed as a target for anti-obesity drugs [63]. Studies have shown that knockdown of *NPY* expression in the hypothalamus increases energy expenditure [64] and overexpression within the paraventricular nucleus results in obesity via increased food intake [65]. We identified a positive association between DNA methylation in *HOXA5* and BMI (nominal p = 2.64×10^−2^), but not waist circumference. *HOXA5* has been implicated in body fat distribution, where it is more highly expressed in visceral (i.e. abdominal) adipose tissue as opposed to subcutaneous (i.e. gluteal) adipose tissue [66], [67]. Further, *HOXA5* is up-regulated in abdominal subcutaneous adipose tissue following fat loss (one year after bariatric surgery) [68]. In addition, nominal associations were seen between either waist circumference or BMI and DNA methylation within several other genes previously implicated in serum triglyceride levels (*ARHGAP9* [69]); obesity and adipogenesis (*CDKN2A* (*p16*) [70], [71], *FRZB* [72], *JAK3* [73], *PEG3* [74]); and glucose homeostasis and insulin resistance (*CDKN2A* [75]–[77], *SMARCB1* [78]).

### Study Limitations

It is important to note the limitations of this study. As both the number of samples (n = 183) and number of CpG sites assessed (n = 395) are small, the power and scope of this study is limited. We have only assayed DNA methylation in one tissue type, peripheral blood, which may limit any generalized conclusions and may not be representative of changes that would be seen in diseased tissue (e.g. adipose tissue). However, given that lipid profiling, glucose measures and cardiovascular-related biomarkers are measured in blood, and that each of these are impacted by obesity, blood represents a good surrogate tissue for these analyses. Many of the results reported here further validate previously reported data and may also offer insight into any ethnic-specific differences.

## Conclusions

We examined the effects of genetic variation, sex and age on DNA methylation and assessed the role of DNA methylation in measures of obesity. Other studies have previously investigated the effects of genetic variation, age and sex using an identical or higher-throughput Illumina platform. However, this is the first study that has examined such factors in a Mexican American population. We have demonstrated here, further evidence supporting a role for genetic effects on DNA methylation, by replicating previous findings and presenting new data. Although several of our findings overlap with previous studies, the identification of novel CpG loci that are heritable, regulated by genetic variation, or that are influenced by age and sex may suggest the existence of ethnic-specific factors that drive regulation of DNA methylation. Such novel findings could also suggest that currently published studies, as well as our own, may be underpowered in their ability to detect true associations or have generated false positive results due to small sample sizes. Differences that are seen across studies could also be due to environmental effects, some of which may themselves be ethnic- or population-specific (e.g. different diets, residential areas). We also provide nominal evidence that DNA methylation may be implicated in obesity in Mexican Americans, however additional follow up studies, particularly in a larger population are required to fully assess such implications.

## Supporting Information

Table S1 Raw data for 188 samples and 1,505 CpG sites.
(ZIP)

Table S2 List of all SNPs within probe sequences.
(XLSX)

Table S3 Heritability estimates for 395 CpG sites assessed.
(XLSX)

Table S4 Complete association analysis between all proximal genetic variants (defined as those within 100 kb) and 393 CpG sites.
(XLSX)

Table S5 Complete association analysis between age and DNA methylation at 395 CpG sites.
(XLSX)

Table S6 Complete association analysis between sex and DNA methylation at 395 CpG sites.
(XLSX)

Table S7 Complete association analysis between obesity measures (waist circumference and BMI) and DNA methylation at 395 CpG sites.
(XLSX)
